# Food Contamination: Major Challenges of the Future

**DOI:** 10.3390/foods5020021

**Published:** 2016-03-23

**Authors:** Malik Altaf Hussain

**Affiliations:** Department of Wine, Food and Molecular Biosciences, Lincoln University, Lincoln 7647, New Zealand; malik.hussain@lincoln.ac.nz

This issue of *Foods* is dedicated to discuss the microbial, chemical and physical contamination challenges of food products. Food contamination is generally defined as foods that are spoiled or tainted because they either contain microorganisms, such as bacteria or parasites, or toxic substances that make them unfit for consumption. A food contaminant can be biological, chemical or physical in nature, with the former being more common. These contaminants have several routes throughout the supply chain (farm to fork) to enter and make a food product unfit for consumption. *Bacillus cereus*, *Campylobacter jejuni*, *Clostridium botulinum*, *C. perfrigens*, Pathogenic *Escherichia coli*, *Listeria monocytogenes*, *Salmonella* spp., *Shigella* spp., Pathogenic *Staphylococcus aureus*, *Vibrio cholera*, *V. parahaemolyticus*, *V. vulnificus* and *Yersinia enterocolitica* are common bacterial hazards (a type of biological contaminant). Chemical food contaminants that can enter the food supply chain include pesticides, heavy metals, and other alien chemical agents. 

The World Health Organization (WHO) has recognized food contamination as a global challenge in several documents and reports [1,2]. It is clearly acknowledged in a statement: “food contamination that occurs in one place may affect the health of consumers living on the other side of the planet” [3]. In fact, a vast majority of people experience a foodborne or waterborne disease at some point in their lives worldwide. Therefore, consumption of contaminated foods causes illness in millions of people and many die as a result of it. This scenario makes “food contamination” a serious issue. The list of food contamination challenges is very long and keeps growing. I would list three challenges, fresh produce contamination, antibiotics in food products and intentional contamination of foods, to highlight the importance of this topic. 

Contamination of fresh produce is emerging as a major food safety challenge. A recent report by the Center for Science in the Public Interest (CSPI) showed that the highest number of outbreaks was attributed to produce as a single commodity in the USA during 2002–2011 [4]. Similarly, produce caused the greatest number of illnesses and the largest average number of illnesses per outbreak. This is a global trend and can be seen in examples of recent outbreaks: an outbreak of *E. coli* O157:H7 after eating contaminated packaged baby spinach in the EU (2006); *E. coli* in cucumber outbreak in Germany and other EU countries (2011); an outbreak of *Cryptosporidium* infection traced to bagged salads in the UK (2012); an outbreak of *L. monocytogenes* due to contaminated prepacked salad products (2016), and a *Salmonella* outbreak linked to lettuce in pre-packaged salads in Australia (2016). 

The emergence of antibiotic resistance bacteria (ARB) is now accepted a potential threat to both public and environmental health and the WHO has already proposed a global strategy to address the challenge [5]. Publications describing the association and prevalence of ARB in food products are common now. Previously the clinical arena was the major culprit; however, the overuse of antibiotics in food production is making the situation more complicated. In brief, foods contaminated with ARB are going to be a major food safety issue in the future.

Intentional contamination of foods and food products is also a growing global concern. Intentional food contamination refers to the deliberate addition of a harmful or poisonous substance to food products. It is a criminal act and also known as food fraud. Foods that have been intentionally contaminated are unsafe to eat and can make consumers seriously ill. Therefore, it is also equally important to address the challenge of fraudulent food contamination.

Finally, I would emphasize that ensuring the supply of safe food products is important to protect public health and the food industry. Food safety is generally compromised when food products get contaminated with a potentially hazardous and toxic agent. The food industry faces many global, as well as regional, contamination issues, existing and emerging, at all times, and continues to address them through scientific and technological developments. Therefore, it is vital for food safety management to understand the nature of contamination, its sources, risks to the consumer, and approaches to eliminate or reduce contamination levels. Sound scientific knowledge is needed to provide food products that are free of contamination or with a minimal risk of contamination This Special Issue contains articles covering food contamination issues, challenges and the solutions.

## References

[1] WHO estimates of the global burden of foodborne diseases—Foodborne diseases burden epidemiology reference group 2007–2015. http://www.who.int/foodsafety/publications/foodborne_disease/fergreport/en/.

[2] Fukuda K. (2015). Food safety in a globalized world. Bull. WHO.

[3] 10 facts on food safety. http://www.who.int/features/factfiles/food_safety/en/.

[4] CSPI (2014). Outbreak Alert! 2014.

[5] Pruden A.R., Pei T., Storteboom H., Carlson K.H. (2006). Antibiotic resistance genes as emerging contaminants: studies in Northern Colorado. Environ. Sci. Technol..

